# Supplementary material for: Identification of photoperiod-induced specific miRNAs in the adrenal glands of Sunite sheep (Ovis aries)
Source: Front Vet Sci. 2022 Jul 22;9:888207. doi: 10.3389/fvets.2022.888207 (PMC9354845; doi:10.3389/fvets.2022.888207)
Supplement: Supplementary file 1 [file Data_Sheet_1.ZIP › Supplementary file/miRDeep2 file.zip!/miRDeep2 file/Know_Structure/SPLP42A3/oar-mir-496.pdf]

miRBase precursor : oar-mir-496
Total read count : 8
oar-miR-496-5p read count : 1
oar-miR-496-3p read count : 6
remaining reads : 1

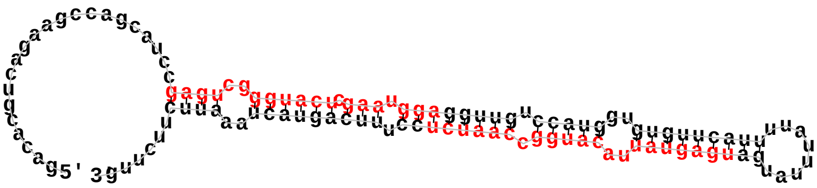

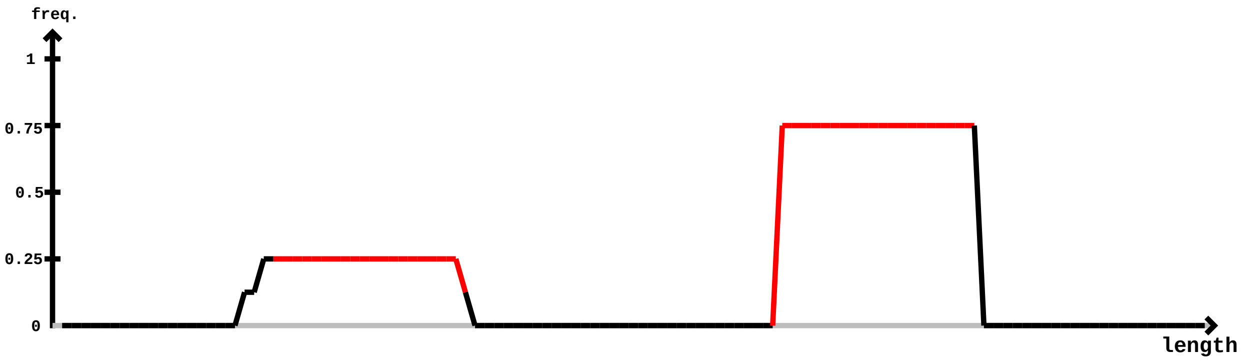


```
                                                                         oar-miR-496-3p
                         oar-miR-496-5p
5'- gacacgucagaagccagcauccgagucggguacucgaauggagguuguccaugguguguucauuuuauuuaugaugaguauuacauggccaaucuccuuucaguacuaaauucuucuug   -3'    exp
    ......................((((..((((((.(((.((((((((.(((((..(((((((((........)))))))))..))))).)))))))).)))))))))..)))).......   reads      mm         sam
    ..................auccgagucggguacucgaaugga.............................................................................   1          0          sec
    ....................ccgagucggguacucgaaugg..............................................................................   1          0          sec
    ..........................................................................ugaguauuacauggccaaucu........................   6          0          sec
```
